# The correlation between latent types of emotional processing, physical exercise and aggressive behavior among middle school students: a study based on latent profile analysis

**DOI:** 10.3389/fpsyg.2026.1815526

**Published:** 2026-04-13

**Authors:** Jie Chu, Yang Yang, Jingtao Wu

**Affiliations:** 1 College of Physical Education, Chengdu Sport University, Chengdu, China; 2 School of Physical Education, Leshan Normal University, Leshan, China

**Keywords:** aggressive behavior, emotional processing type, latent profile, middle school students, physical exercise

## Abstract

**Objective:**

From the perspective of latent profile analysis (LPA), this study explored the relationships among emotional processing types, physical exercise, and aggressive behavior of middle school students, and clarified the mechanism of physical exercise in the association between emotional processing types and aggressive behavior.

**Method:**

A cross-sectional survey was conducted in multiple regions of six provinces and municipalities in China from October to December 2025. By adopting the method of random cluster sampling, a total of 7,276 middle school students were recruited as research subjects. The Emotion Regulation Questionnaire, Physical Exercise Rating Scale, Anger Rumination Scale and Aggressive Behavior Scale were used for measurement, and *SPSS* 31.0 and *Mplus* 8.3 software packages were applied for data analysis.

**Results:**

(1) There were four distinct latent types of emotional processing among middle school students, namely adaptive emotional processing type, high rumination-inhibition emotional processing type, mixed emotional processing type, and reflection-dominated emotional processing type. (2) There were significant differences in aggression and physical exercise levels across different types of emotion processing. The adaptive emotion processing type exhibited the lowest aggression and the highest physical exercise level, while the reflection-dominant type showed the highest aggression and the lowest physical exercise level, indicating a trend of lower aggression levels being associated with higher physical exercise participation. (3) After controlling for demographic variables, physical exercise and emotional processing types could significantly predict aggressive behavior; anger rumination and expressive suppression were identified as risk factors for increased aggressive behavior, while cognitive reappraisal and physical exercise served as protective factors that curbed aggressive behavior.

**Conclusion:**

Physical exercise is significantly negatively correlated with adolescent aggressive behavior, and different types of emotional processing show differences in aggression risk and physical exercise levels. The results provide empirical evidence for stratified interventions based on emotional processing types, while causal relationships require further longitudinal tracking and experimental verification.

## 1 Introduction

Adolescence is a critical period for personality shaping and a key window for the development and consolidation of social behavior patterns (Nickerson et al., 2026). In recent years, campus conflicts, peer conflicts, verbal bullying and cyber aggression have become increasingly common, making the aggressive behavior of middle school students a focal issue on campus and a hot topic of widespread concern in society (David-Ferdon et al., 2023). Scholars have pointed out that simply defining aggressive behavior as a result of conflict or moral deficiency fails to clearly distinguish individual differences (Fite et al., 2022). In the context of campus aggressive behavior triggered by conflicts or provocations, some students can remain restrained, while others quickly engage in confrontational conflicts. Scholars argue that aggressive behavior is not a simple isolated incident, but a product of the interaction between an individual’s internal emotional processing and external emotional exchanges (Gross, 2013). To address the social phenomenon of aggressive behavior among middle school students, clarifying the underlying mechanisms of emotional processing is the fundamental solution (Wang L. et al., 2024). Therefore, against the backdrop of building a strong educational nation and a society based on the rule of law, paying attention to the psychological characteristics of adolescent aggressive behavior is a major issue that urgently needs to be addressed by the times.

In this study, emotion processing refers to relatively stable regulatory patterns among adolescents in negative emotional contexts (Gross, 2013), encompassing three combinations of strategies involving cognitive, expressive suppression, and anger rumination. We focus on the co-occurrence patterns of these strategy combinations within individuals, as well as the associations between different types of strategy and physical exercise and aggressive behavior. In the development of individual psychological characteristics, emotion is not a single subjective feeling of an individual, but a profound and complex system involving emotional evaluation, expression and reflection. An individual’s definition of a situation, expression strategies, and tendency to ruminate on emotions all directly influence the development of behavior. Some adolescents alleviate negative emotions through cognitive reappraisal, some choose expressive suppression to maintain superficial harmony (Li et al., 2025), and others indulge in emotional rumination, which accumulates negative emotions (Everaert and Joormann, 2020). These different coping strategies are not simply a ranking of advantages and disadvantages, but stable emotional manifestations of an individual’s overall external behavior (Bonanno and Burton, 2013). It is precisely these structural differences in strategies that lead to variations in the risk of aggressive behavior. Scholars have found that previous studies mostly focused on single variables to explore the impact of a certain emotion regulation strategy on aggressive behavior, ignoring the potential types and changing patterns within emotional processing (Navas-Casado et al., 2023). In reality, adolescent aggressive behavior does not simply show a high or low distribution, and may be associated with underlying psychological disorders (Squillaci and Benoit, 2021). Without an in-depth and dialectical analysis of the types of psychological characteristics, relevant conclusions will be difficult to accurately serve the public and effectively support targeted interventions.

As an intervention method that benefits physical health, physical exercise has gradually been recognized by the public as an important factor affecting the mental health development of adolescents (World Health Organization, 2020). Participation in daily physical activities involves the inculcation of rule awareness, the cultivation of a cooperative atmosphere, and the awakening of physical regulation awareness, which inherently plays an important role in shaping an individual’s emotional processing and prompting individuals to adopt more mature coping styles when facing setbacks (Eime et al., 2013). Different from previous pure psychological interventions, physical exercise shapes behavioral habits through practical activities, which are accompanied by the formation of behavioral habits and rooted in subsequent life (Hawlader et al., 2023). Scholars have not yet reached a consistent conclusion on whether physical exercise directly affects an individual’s aggressive behavior or exerts its influence through the mediation of emotion regulation strategies (Wang et al., 2025). Based on the above research, this study introduced the latent profile analysis method on the basis of previous scholars’ work to explain the internal structural characteristics of emotional processing among middle school students, and examine the differences in aggressive behavior and physical exercise among different latent types. On this basis, it further explored the predictive effects and correlation mechanisms of physical exercise and emotional processing types on aggressive behavior, aiming to provide theoretical reference and empirical evidence for hierarchical and targeted treatment and educational intervention of adolescent aggressive behavior.

### 1.1 Overview of the psychological mechanisms of adolescent aggressive behavior

Adolescent aggressive behavior is a hot topic in developmental psychology and social development research. With the in-depth exploration and advancement of scholars, its connotation has evolved from the traditional focus on behavioral facts to the investigation of psychological mechanisms. In early studies, scholars defined aggressive behavior from the perspective of behavioral consequences, regarding it as hostile and harmful physical or verbal behavior toward others, and emphasizing its external behavioral characteristics (Lakens, 2025). Scholars argue that aggressive behavior is not a single incident, but may be an individual’s emergency response to threats in specific situations (Allen and Anderson, 2017). Therefore, in the conceptual definition, aggressive behavior is mainly distinguished by its different external manifestations: overt aggressive behavior refers to direct physical or verbal conflicts, while relational aggressive behavior refers to behaviors that cause harm to others by excluding, isolating and destroying their interpersonal networks (Van Bockstaele, 2024). Scholars have basically defined the manifestations of aggressive behavior and explained the differences in individual social functions, such as impulsive outbursts or strategic regulation. In the study of psychological mechanisms, aggressive behavior is the result of the interaction of individual emotional arousal, hostile cognition and behavioral decision-making, accompanied by the occurrence of anger, hostility and impulsive behavior (Huseynov and Ozdenizci Kose, 2024). Therefore, the research focus of scholars has gradually shifted from “what happened” to “why it happened”.

Scholars have gradually formed a relatively systematic explanatory framework around the exploration of the mechanisms and theories of aggressive behavior. The General Aggression Model emphasizes that individual factors (personality traits, beliefs, will) and external situational factors (external stimuli, social conflicts, setbacks, rumination) jointly act on an individual’s internal emotional system, affecting emotional experience, cognitive processing and physiological emotional arousal. This internal state of an individual is processed through emotional processing, ultimately leading to aggressive or non-aggressive behavior (Din and Ahmad, 2021). The model holds that an individual’s aggressive behavior is a cyclical process rather than a single social event, and emphasizes that the continuous accumulation of social cognitive level can curb or reduce the occurrence of aggressive behavior (Hagger and Hamilton, 2024). The Emotional Arousal Theory holds that individuals with high arousal levels may not necessarily engage in aggressive behavior, but their window of rational evaluation will be reduced, making impulsivity more likely to be triggered (Sang et al., 2026). Therefore, there is a correlation between arousal intensity and emotion regulation, which is an important condition affecting the risk of aggressive behavior (Басюк, 2025). At the same time, scholars of the Self-Control Theory argue that whether an individual has regulatory resources or the conditions for expressive suppression after a conflict occurs is crucial (Pusch, 2025). Insufficient self-control ability is likely to weaken the function of emotion regulation, leading to aggressive behavior breaking through rational constraints (Wang W. et al., 2024). All the above scholars have discussed different paths of aggressive behavior, and all point to the fact that the occurrence of aggressive behavior is the result of the interaction of emotional processing, cognitive reappraisal and emotional control, rather than the influence of a single factor.

Although scholars’ theories have been continuously evolving, empirical studies have mainly tended to adopt a variable-centered research approach, mainly observing the predictive effects of certain variables on aggressive behavior. Although these strategies help to explain the overall development trend, they also obscure the differences among adolescent groups (Woo et al., 2024). In fact, different individuals have different emotional processing styles: some individuals with high emotional arousal tend to use cognitive reappraisal, while others prefer expressive suppression to curb negative emotions. These differences jointly constitute multiple paths of emotional processing and are the main sources of the risk of aggressive behavior. If research only stays at the level of linear relationship analysis, it is difficult to answer the practical question of which psychological patterns are more risky. Therefore, it is necessary to break through the framework of single structural variables, analyze the emotional processing system as a whole from a structural perspective, and sort out potential psychological types to explain the differentiated paths behind aggression, which is conducive to the subsequent development of targeted psychological interventions.

### 1.2 Structural characteristics and consequences of emotional processing strategies

In the current emotional processing framework, emotion generation is not an instantaneous subjective reaction of an individual, but a systematic process involving situational evaluation, expression awareness and post-event reflection. When facing external stimuli, individuals evaluate and reappraise the situation and then review and reflect on the emotional experience. Scholars believe that cognitive reappraisal is a regulation method that weakens the expression of negative emotions from the source by reinterpreting and understanding the situation (Sychenko et al., 2025). Expressive suppression is a post-hoc individual reaction, which refers to the external suppression and restraint behavior of an individual after the generation of emotion (Caramanica et al., 2023). Emotional rumination refers to the reflection and in-depth thinking of past negative experiences, which makes individuals indulge in a vicious circle of negative emotional processing (Wong et al., 2023). These three strategies have significant differences in function and psychological expression paths: cognitive reappraisal helps individuals maintain psychological resilience, expressive suppression may lead to the accumulation of negative emotions, and emotional rumination is regarded as a major potential risk factor (Cludius et al., 2020). Importantly, these strategies do not exist in isolation, but may coexist or combine within individuals, forming relatively stable emotional processing patterns.

At the behavioral level, differences in adolescents’ emotional processing strategies are associated with different aggressive tendencies. Cognitive reappraisal can reduce hostility and emotional fluctuations when facing provocations and setbacks, thereby lowering anger and impulsive behavior (Pelz, 2025); it occurs in the early stage of emotion generation and reduces the probability of negative emotion accumulation and its transformation into aggressive behavior (Lavallee et al., 2025). In contrast, although expressive suppression can curb overt impulsive behavior, it cannot eliminate the source of emotion (Hosie et al., 2022), and long-term accumulation may increase the probability of conflict outbursts. Emotional rumination may intensify negative emotions and hostile cognition, increasing the arousal level and likelihood of aggressive behavior (Pop et al., 2025). Therefore, the three strategies differ in their potential behavioral consequences, and their combinations may shape heterogeneous risk levels of aggressive behavior.

Methodologically, previous studies mainly used structural equation modeling or regression to test the effect of a single strategy (Howard and Hoffman, 2018), which may overlook how multiple strategies co-occur within individuals. In practice, adolescents may simultaneously show varying levels of cognitive reappraisal, expressive suppression and emotional rumination, suggesting diverse configurations (Cho et al., 2025). Therefore, a person-centered latent profile analysis is needed to identify emotional processing types from the perspective of group structure and to compare their differentiated behavioral risks.

### 1.3 Intervention value of physical exercise in emotional processing and aggressive behavior

With the in-depth development of adolescent emotional processing, physical exercise is no longer simply a physical activity, but an embedded emotion regulation strategy that plays an important role in daily life. Scholars believe that the effects of physical exercise on adolescent psychological counseling and emotional processing are mainly reflected in physiological regulation. It is generally believed that regular physical activity can arouse the individual’s nervous system and emotions, promote the balance of individual endocrine, alleviate stress and setbacks in daily life, and maintain a relatively stable emotional state and physiological function of individuals (Biddle et al., 2019). Compared with passive emotion regulation methods, physical exercise encourages individuals to participate and experience changes in physical state, thereby improving the ability to regulate emotions. Furthermore, physical exercise is a competitive activity with a strong sense of rules, which incorporates elements such as delayed gratification and team cooperation. In previous physical exercise activities, individuals unconsciously promote the stability of self-regulation by abiding by rules, controlling impulsivity, and accepting external setbacks, which provides more regulatory space for individuals before the arousal of impulsivity (Ruiz-Ranz and Asín-Izquierdo, 2025). Physical activity is also an important window for emotional catharsis. Participation in daily physical exercise brings more positive benefits, relieves tension, discomfort, negative emotions and stress, and avoids their transformation into aggressive behavior (Wu et al., 2025). Physical exercise is not a simple physical activity, but an interactive deployment of physiological, psychological and social interaction dimensions, bringing the exchange of benefit value.

With the gradual development of physical exercise in intervening emotion regulation, scholars believe that physical exercise is an important means to shape an individual’s emotional processing and plays a crucial role in intervening aggressive behavior (Hu and Liu, 2025). At the cognitive level, persistent participation in physical activity helps individuals develop good living habits and shape positive emotions (Gressieux and Meunier, 2024). When facing external social stimuli, setbacks and failures, the benefits brought by exercise will be transformed into the perception of effort or strategic problems, avoiding hostility or the sense of unfairness, and bringing the reshaping of cognitive value and the improvement of emotional value (Mschwelitze, 2024). Reflection and emotional adjustment in the context of physical exercise provide a field and environment for redefining emotional value. At the same time, regular participation in physical exercise can curb the reflection of negative emotions and the occurrence of emotional rumination, reduce the thinking and indulgence in negative emotional events, and further inhibit the occurrence of emotional rumination (Sürü, 2022). Compared with the long-term emotional internal friction in a static environment, physical exercise brings a new development perspective, provides more adjustment space and emotional value, and prompts individuals to break away from emotional predicaments (Hasani et al., 2025). For expressive suppression, physical exercise provides a broader space for expression and a release field. Within the scope permitted by rules and morality, individuals can release inner depression and emotional discomfort, thereby alleviating emotional problem behaviors (Antuña-Camblor et al., 2024). Physical exercise not only directly acts on aggressive behavior, but may also reshape the expression path of conflict occurrence by changing the emotional processing pattern.

However, current research mainly stays at the level of linear relationship between physical exercise and aggressive behavior, focusing on the effect size, expression path and regulatory mechanism, and few studies explore the mediating mechanism of emotional processing (López-Pernas et al., 2025). Among adolescent groups, there is a lack of research on physical exercise reshaping emotional processing patterns and changing the expression of individual problem behaviors, and theoretical research is relatively scarce. These series of studies cannot be systematically answered in variable-centered research. Although some current studies explore the relationship between personality and behavior, research on emotional processing and physical exercise is still scarce, and empirical research based on latent profile analysis is particularly insufficient. If physical exercise is regarded as an important external variable affecting emotional processing, and the latent profile analysis method is used to identify different psychological models, it will help to reveal the real structure of risk stratification of aggressive behavior and lay a solid foundation for the subsequent development of psychological intervention work.

### 1.4 Research hypotheses

Based on previous research, emotion regulation strategies are closely related to rumination and adolescent internalizing and externalizing problem behaviors; individual differences in emotion regulation strategies manifest combination patterns, and physical exercise is associated with emotion regulation and problem behaviors. Based on this, this study puts forward the following research hypotheses: H1: There are multiple latent types of emotional processing among middle school students; H2: There are significant differences in the level of aggressive behavior among different emotional processing types; H3: There are significant differences in the level of physical exercise among different emotional processing types; H4: After controlling for demographic variables, physical exercise and emotional processing types can significantly predict aggressive behavior.

## 2 Research subjects and methods

### 2.1 Research subjects

**Figure 1:**
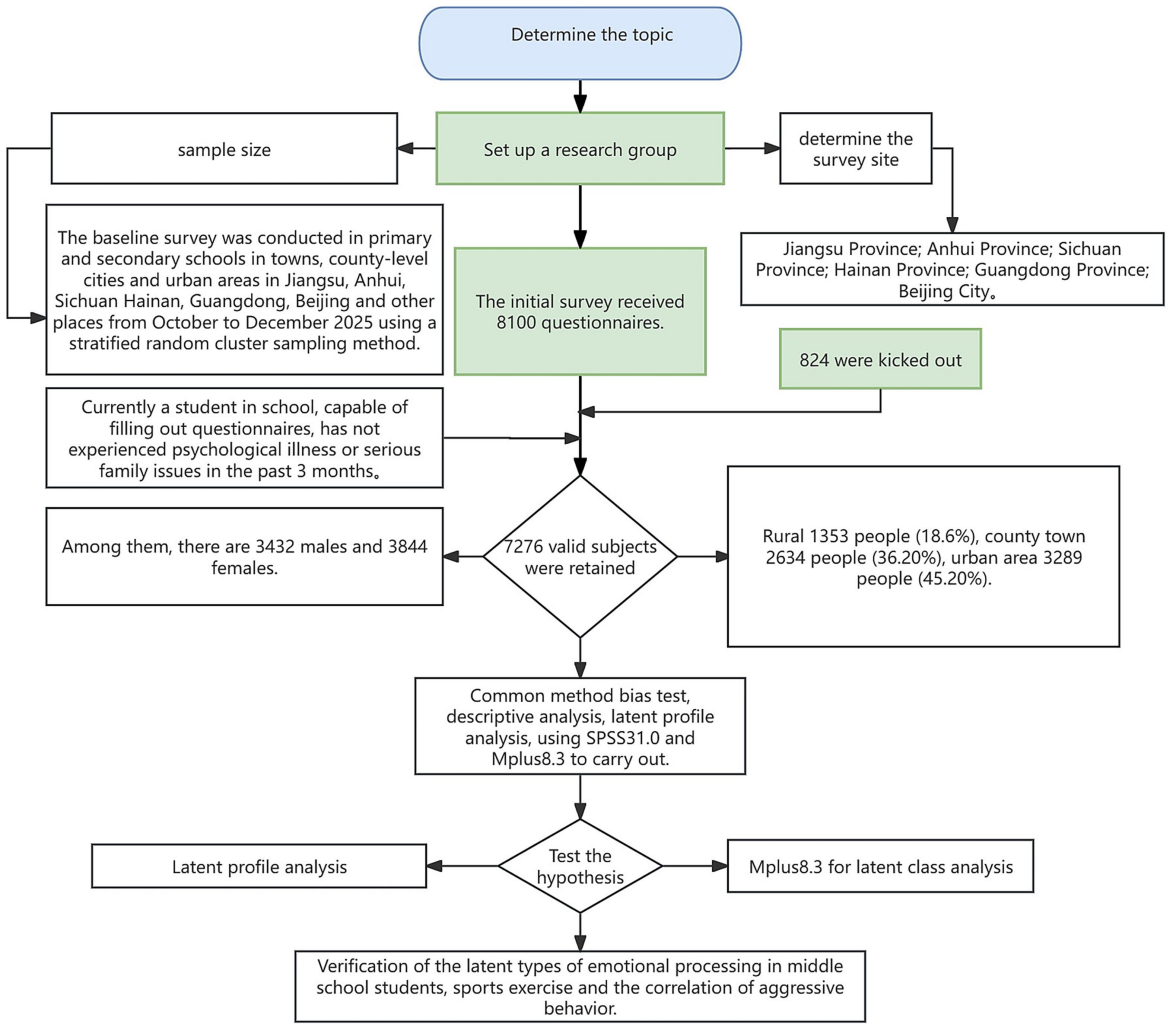
The research framework.

This study adopted the method of random cluster sampling to recruit middle school students from Chengdu and Leshan in Sichuan Province, Suzhou and Yancheng in Jiangsu Province, Guangzhou and Heyuan in Guangdong Province, Sanya and Haikou in Hainan Province, Beijing, Hefei and Huainan in Anhui Province, and other regions. Students in Grades 1 to 3 of junior high school and senior high school were selected as the research subjects. The study was launched in early October 2025, the questionnaires were distributed by the research teams in various regions, and the basic questionnaire survey was completed in December 2025. This research protocol was approved by the Ethics Committee of the Academic Committee of Leshan Normal University (Approval No.: LSNU:1037-26-01RO). The research subjects were required to meet the following conditions: currently enrolled in school, capable of filling out the questionnaire, and no psychological disorders or serious family problems in the past 3 months. This study adopted a centralized administration approach, providing standardized instructions and on-site clarification to ensure that participants could understand and complete the corresponding questionnaires. A total of 8,100 questionnaires were distributed, and 7,276 valid questionnaires were retained after excluding invalid ones, including 3,432 from boys and 3,844 from girls. The specific research framework is shown in Figure 1.

### 2.2 Research methods

#### 2.2.1 Emotion regulation questionnaire

The Emotion Regulation Questionnaire compiled by Gross and John (2003) and revised and localized by Wang et al. (2007) was used to systematically measure the emotion regulation ability of middle school students. The questionnaire includes two dimensions, namely cognitive reappraisal and expressive suppression, with a total of 10 items (6 for cognitive reappraisal and 4 for expressive suppression). A 7-point Likert scale was used for evaluation, with scores ranging from 1 (completely inconsistent) to 7 (completely consistent). The score of each dimension is the sum of the scores of each item, with higher scores indicating a higher frequency of using the corresponding emotion regulation strategy. The Cronbach’s *α* coefficient of the questionnaire was 0.86, and those of cognitive reappraisal and expressive suppression were 0.88 and 0.84, respectively.

#### 2.2.2 Physical exercise rating scale (PARS-3)

The Physical Exercise Rating Scale compiled and revised by Liang (1994) was used to measure the basic situation of the subjects’ participation in physical exercise in the past week. The scale consists of three items, including the intensity, duration and frequency of physical exercise. A 5-point Likert scale was used for evaluation, with higher scores indicating a higher level of physical exercise participation. The Cronbach’s *α* coefficient of the scale was 0.83, showing good construct validity.

#### 2.2.3 Buss-Perry aggression questionnaire (BPAQ)

The Buss-Perry Aggression Questionnaire compiled by Buss and Perry (1992) and revised and localized by Chinese scholar Liu et al. (2009) was used. The scale includes four dimensions, namely physical aggression, anger, hostility, and displaced aggression, with a total of 20 items. A 5-point Likert scale was used for measurement, with higher scores indicating a higher level of individual aggression. The Cronbach’s *α* coefficient of the scale was 0.82, and those of the sub-dimensions were 0.83, 0.79, 0.84 and 0.86, respectively, showing good validity for both the whole scale and sub-dimensions.

#### 2.2.4 Ruminative response scale (RRS)

The Chinese version of the Ruminative Response Scale compiled by Han and Yang (2009) was used. The scale includes three dimensions, namely symptom rumination, obsessive thinking, and reflective pondering, with a total of 22 items. A 4-point Likert scale was used for evaluation, with scores ranging from 1 (occasionally) to 4 (always). Higher scores indicate more severe ruminative thinking. The Cronbach’s *α* coefficient of the scale was 0.81, and those of the sub-dimensions were 0.83, 0.79, and 0.82, respectively, showing good overall structural reliability.

### 2.3 Data analysis

SPSS 31.0 and Mplus 8.3 software packages were used for data analysis in this study. First, the four scales were standardized to avoid systematic biases caused by different Likert scale levels (Sinha et al., 2021). Descriptive analysis was conducted to calculate the overall distribution trend and characteristics of the sample. Pearson correlation coefficient was used to determine the significance and magnitude of the correlation between variables. Harman’s single-factor test was adopted to examine the common method bias (Fuller et al., 2016). Mplus 8.3 was used for latent profile analysis, comparison of fitting indexes (Nylund-Gibson and Choi, 2018), probability calculation and difference comparison. The BCH method was used to compare the effect differences (Lê et al., 2025). Finally, SPSS was used for regression analysis to compare the differences under different variable indicators. A *p* value of < 0.05 was considered statistically significant.

## 3 Results and analysis

### 3.1 Common method Bias test

To avoid systematic biases caused by the same respondent method, Harman’s single-factor variance test was used to extract the common factor variance of all scales (Fuller et al., 2016). The results showed that there were five characteristic roots greater than 1, and the explained variance of the largest common factor was 26.31%, which was lower than the critical standard of 40%, indicating that there was no serious common method bias in this study.

### 3.2 Descriptive statistics and correlation analysis

As shown in Table 1, a total of 7,276 subjects were included in this study, including 3,844 females (52.83%) and 3,432 males (47.17%). The number of students in the 7th and 8th grades of junior high school was relatively large, accounting for 25.45 and 28.38%, respectively. In terms of boarding status, 2,448 students (33.64%) lived on campus and 4,828 students (66.36%) did not. In terms of place of origin, the number of students from rural areas was relatively small (1,353, 18.60%), followed by those from county towns (2,634, 36.20%) and urban areas (3,289, 45.20%). In terms of family type, 990 students (13.61%) came from single-parent families, 5,887 (80.91%) from two-parent families, and 399 (5.48%) from other family types. On the whole, the demographic variables were relatively evenly distributed.

**Table 1:** Basic information of demographic variables (*N* = 7,276).

Variable	Category	Frequency	Percentage (%)	Cumulative percentage (%)
Grade	7th grade	1,852	25.45	25.45
	9th grade	942	12.95	38.40
	8th grade	2,065	28.38	66.78
	10th grade	784	10.78	77.56
	12th grade	1,026	14.1	91.66
	11th grade	607	8.34	100
Gender	Female	3,844	52.83	52.83
	Male	3,432	47.17	100
Boarding status	No	4,828	66.36	66.36
	Yes	2,448	33.64	100
Place of origin	Rural area	1,353	18.6	18.60
	County town	2,634	36.20	54.80
	Urban area	3,289	45.20	100
Family type	Other	399	5.48	5.48
	Single-parent	990	13.61	19.09
	Two-parent	5,887	80.91	100
Total		Total	100	100

As shown in Table 2, physical exercise was significantly negatively correlated with expressive suppression, emotional rumination and aggressive behavior, with correlation coefficients ranging from −0.214 to −0.270, and significantly positively correlated with cognitive reappraisal. Cognitive reappraisal was significantly negatively correlated with expressive suppression, emotional rumination, and aggressive behavior, with correlation coefficients ranging from −0.281 to −0.617. Expressive suppression, emotional rumination, and aggressive behavior were significantly positively correlated with each other, with correlation coefficients ranging from 0.294 to 0.521 (all *p* < 0.001).

**Table 2:** Correlation analysis of physical exercise, emotional processing indicators and aggressive behavior (*N* = 7,276).

Variable	M	SD	1	2	3	4	6
1. Physical exercise	3.185	0.963	1				
2. Cognitive reappraisal	3.224	1.066	0.453**	1			
3. Expressive suppression	3.436	0.864	−0.247**	−0.281**	1		
4. Emotional rumination	3.161	0.99	−0.214**	−0.464**	0.521**	1	
5. Aggressive behavior	3.009	1.035	−0.270**	−0.617**	0.294**	0.369**	1

M, mean; SD, standard deviation; *p* < 0.001.

### 3.3 Latent profile analysis of emotional processing among middle school students

As shown in Table 3, the fitting indexes of the latent profile analysis (LPA) of emotional processing included AIC, BIC, aBIC and other relevant indexes. The AIC and BIC values of the 2-class model were 328747.465 and 329064.512 respectively, showing poor fitting, and the Entropy value was 0.871 with an unbalanced overall classification. The 3-class and 4-class models showed better fitting indexes with a significant improvement compared with the 2-class model, while the 5-class model had higher fitting indexes and a higher Entropy value, indicating a higher probability of hierarchical classification. However, considering the overall distribution characteristics and fitting indexes of the model, the 4-class model was the optimal one. This verified that there were obvious latent types of emotional processing among middle school students, thus supporting Research Hypothesis H1.

**Table 3:** Comparison of fitting indexes of latent profile models of emotional processing.

Variables	Model	K	Log(L)	AIC	BIC	aBIC	Entropy	LMR*p*	BLRT*p*	Probabilistic category
Emotional processing model	2C	46	−164327.732	328747.465	329064.512	328918.334	0.871	<0.001	<0.001	0.574/0.424
	3C	62	−160059.825	320243.651	320670.975	320473.953	0.863	<0.001	<0.001	0.266/0.462/0.271
	4C	78	−156160.16	312476.32	313013.922	312766.055	0.871	<0.001	<0.001	0.366/0.222/0.1970.215
	5C	94	−154099.603	308387.206	309035.086	308736.375	0.879	<0.001	<0.001	0.154/0.372/0.180/0.144/0.151

K, Number of latent classes; Log(L), Log-likelihood; AIC, Akaike Information Criterion (the smaller the better); BIC, Bayesian Information Criterion (the smaller the better); aBIC, Adjusted Bayesian Information Criterion (the smaller the better); Entropy, Classification accuracy index (the closer to 1 the better); LMR p, LMR test *p*-value (K classes vs K − 1 classes); BLRT p, BLRT test *p*-value (K classes vs K − 1 classes); Probabilistic category, Proportion of samples in each class/Posterior probability.

As shown in Table 4, the classification probabilities of the latent types of emotional processing were the assignment probabilities of each type. The classification probabilities of C1, C2, C3, and C4 were 0.916, 0.929, 0.955, and 0.909 respectively, with most samples having a high probability of being assigned to their respective classes, indicating a high classification resolution. In summary, there are identifiable latent structural types of emotional processing among middle school students, which further verifies Research Hypothesis H1.

**Table 4:** Classification probability matrix of latent types of emotional processing.

Variables	Category	C1 (%)	C2 (%)	C3 (%)	C4 (%)
Emotional processing model	C1	0.916	0.030	0.020	0.034
	C2	0.053	0.929	0.000	0.018
	C3	0.034	0.000	0.955	0.011
	C4	0.062	0.016	0.013	0.909

As shown in Table 5, there were significant differences in aggressive behavior and physical exercise among different latent types of emotional processing. The mean aggression score was lowest for the adaptive emotion processing type (Class 1) and highest for the reflection-dominant emotion processing type; regarding physical exercise, the adaptive emotion processing type had the highest participation level, while the reflection-dominant type had the lowest. The Wald test results showed that the overall differences in aggressive behavior and physical exercise were significant, with Wald χ^2^ values of 3866.898 (*df* = 3, *p* < 0.001) and 1301.841 (*p* < 0.001) respectively, indicating that there were significant differences in the levels of aggressive behavior and physical exercise among different emotional processing types. Thus, Research Hypotheses H2 and H3 were verified.

**Table 5:** Differences in aggressive behavior and physical exercise among different latent types of emotional processing.

Emotional processing type	Aggressive behavior (M, SE)	Physical exercise (M, SE)
Adaptive emotional processing type (Class 1)	1.691 (0.032)	3.681 (0.022)
High rumination-inhibition emotional processing type (Class 2)	2.820 (0.032)	3.553 (0.026)
Mixed emotional processing type (Class 3)	2.189 (0.029)	2.981 (0.018)
Reflection-dominated emotional processing type (Class 4)	4.378 (0.036)	2.591 (0.027)

M, Mean; SE, standard error.

As shown in Table 6, there were significant differences in the levels of aggressive behavior and physical exercise among different latent types of emotional processing, with all Wald test results being significant (all *p* < 0.001). The adaptive emotional processing type (Class 1) was significantly different from other types in both aggressive behavior and physical exercise, indicating that lower levels of aggressive behavior were associated with higher levels of physical exercise participation. Among them, the difference between Class 1 and Class 4 was the most significant in behavioral performance and physical exercise level, followed by the difference between Class 2 and Class 3. Overall, emotion regulation strategies showed a diversified trend among adolescents and significantly affected behavioral performance and physical exercise participation levels. The significant differences in behavioral performance and physical exercise among different emotional processing types further confirm that Research Hypotheses H2 and H3 are valid.

**Table 6:** Pairwise comparisons of aggressive behavior and physical exercise among latent types of emotional processing (BCH method).

Comparison	Aggressive behavior		Physical exercise	
	Wald χ^2^	*p*	Wald χ^2^	*p*
Class 1 vs. Class 2	933.541	<0.001	13.937	<0.001
Class 1 vs. Class 3	2300.524	<0.001	553.826	<0.001
Class 1 vs. Class 4	3109.219	<0.001	1001.381	<0.001
Class 2 vs. Class 3	172.789	<0.001	299.458	<0.001
Class 2 vs. Class 4	657.451	<0.001	660.580	<0.001
Class 3 vs. Class 4	107.090	<0.001	137.561	<0.001

Wald χ^2^, Test statistic for the mean difference between two classes; P, Significance level (the smaller the value, the more significant the difference).

### 3.4 Predictive effects of physical exercise and emotional processing on aggressive behavior

As shown in Table 7, after controlling for demographic variables, the regression model explained 63.6% of the variance in aggressive behavior, showing a high predictive rate. From the perspective of the *β* values and significant *p* values of the observed variables, physical exercise had a significant negative predictive effect on aggressive behavior (*β* = −0.107, *p* < 0.001); cognitive reappraisal also had a significant negative predictive effect (*β* = −0.253, *p* < 0.001), while expressive suppression (*β* = 0.198, *p* < 0.001) and anger rumination (*β* = 0.314, *p* < 0.001) had significant positive predictive effects on aggressive behavior. Among the demographic variables, grade and boarding status had significant predictive effects: students in higher grades and those living in urban areas tended to have lower levels of aggressive behavior. On the whole, physical exercise and emotion regulation strategies (cognitive reappraisal and anger rumination) are key factors predicting adolescent aggressive behavior. That is, after controlling for demographic variables, physical exercise and emotional processing can still significantly predict aggressive behavior, thus verifying Research Hypothesis H4.

**Table 7:** Regression analysis results of physical exercise and emotional processing indicators predicting aggressive behavior.

Independent variable	*β*	*SE*	*Z*	*p*
Grade	−0.048	0.01	−4.745	<0.001
Gender	−0.010	0.009	−1.107	0.268
Boarding status	0.022	0.01	2.341	0.019
Place of residence	0.046	0.009	4.841	<0.001
Family type	−0.003	0.009	−0.299	0.765
Physical exercise → Aggressive behavior	−0.107	0.013	−8.165	<0.001
Cognitive reappraisal → Aggressive behavior	−0.253	0.019	−13.316	<0.001
Expressive suppression → Aggressive behavior	0.198	0.017	11.647	<0.001
Anger rumination → Aggressive behavior	0.314	0.019	16.526	<0.001
Multiple correlation coefficient (R)	0.801			
Coefficient of determination (R^2^)	0.636			

β, standardized regression coefficient; SE, standard error; Z, test statistic; P, significance level; R, multiple correlation coefficient; R^2^, coefficient of determination (proportion of variance explained by the model).

## 4 Discussion

### 4.1 Identification of latent types of emotional processing and model fitting

This study identified the latent types of emotional processing among middle school students through latent profile analysis, and the results showed that the 4-class model had reasonable fitting indexes and interpretability. The data showed that with the increase in the number of classes, indexes such as AIC and BIC decreased successively; the 5-class model had better fitting indexes but a more scattered classification and relatively weak interpretability. Considering the Entropy value, category probability and other indexes of latent profile classification, the 4-class model was more appropriate in line with the principle of parsimony. The data structure presented relatively reasonable distribution characteristics, supporting the view that emotional processing is not a single structure and showing a latent category structure with internal consistency (Sakiris and Berle, 2019). This is slightly different from the viewpoint proposed by Gross, who emphasized the differences in the time course and functional positioning of the process model of emotion regulation, but both pointed out the diversified development mode of individual emotion regulation, namely the multi-class structure of emotional processing patterns (Gruber et al., 2020). The multi-class model of emotional processing identified in this study empirically proves the validity of the theory, indicating that there are structural differences in adolescents’ emotional processing styles, rather than a linear trend of high or low distribution.

At the same time, the latent profile analysis adopted in this study is consistent with the application of the person-centered approach and conforms to the current trends and characteristics of psychological research. A large number of empirical results from previous scholars have proved that the measurement based on item scales is mostly a grade evaluation based on norms, mainly emphasizing the negative correlation between cognitive reappraisal and aggressive behavior and the equivalent relationship between expressive suppression and emotional rumination. The overall research mainly explores linear correlations, which will cover up the internal psychological development characteristics of individuals (Morin et al., 2018). This study identified four emotional processing types with high assignment probability through latent profile analysis, with all category probabilities higher than 0.90, showing good stability and discriminant validity. This result is consistent with the conclusion of multiple types of emotion regulation proposed by foreign scholars (Herman et al., 2024). In addition, this study is not a simple binary regression of outcome variables, but identifies the internal diversified development trends, suggesting that adolescent emotional processing is a complex system. The mutual verification of the research results and theoretical achievements further clarifies that when explaining external problem behaviors such as aggressive behavior (Peri et al., 2024), it is necessary to think from the perspective of structured multi-type development, dialectically view the differences between single strategies and diversified classifications, and avoid falling into the systematic misunderstanding of single empirical research.

### 4.2 Differences in aggressive behavior and physical exercise among different emotional processing types

After identifying the four latent types of emotional processing, this study further explored the differences in aggressive behavior and physical exercise among different types. The results showed that there were significant differences in aggressive behavior and physical exercise among different emotional processing types, with the overall significance level meeting the standard and significant pairwise comparisons. The results confirmed that Research Hypotheses H2 and H3 were valid, that is, there were structural differences in emotional processing types with stable category performance and significant stratification in behavioral performance and physical exercise participation. Overall, the adaptive emotion processing type exhibited lower levels of aggressive behavior and higher levels of physical exercise; the reflective-dominant type showed higher levels of aggression and higher levels of physical exercise, with the most significant difference observed between the first and fourth types. These structural characteristics are consistent with the conclusions of previous studies on the functions of emotion regulation (Brenning et al., 2022). Scholars’ process model of emotion regulation also points out that cognitive reappraisal helps to reduce the expression and disclosure of negative emotions, while rumination and expressive suppression will aggravate the outbreak of problem behaviors, that is, they are positively correlated with aggressive behavior (Ahmad and Oriani, 2023). This study proves this point from the perspective of types: these emotion regulation and processing patterns do not exist in isolation, but are mixed patterns in different individuals, which are ultimately reflected in the expression level of aggressive behavior.

Secondly, the differential expression of physical exercise among different emotional processing types also supplements and improves the existing theories (Fu et al., 2025). Empirical studies have pointed out that physical exercise can enhance an individual’s self-control ability and emotion regulation ability, and reduce the incidence of externalizing problem behaviors such as aggressive behavior (Hochstein et al., 2025). The results of this study are consistent with the conclusions of previous studies. Physical exercise shows significant differences across different types of emotional processing, exhibiting an opposite developmental trend to aggressive behavior; types with lower levels of aggression demonstrate higher levels of physical exercise (Schuler et al., 2014), that is, the more adaptive the emotional processing structure, the higher the motivation and level of physical exercise participation. These conclusions are consistent with the results in Table 2, that is, physical exercise is positively correlated with cognitive reappraisal and negatively correlated with emotional rumination, confirming that physical exercise can be independently embedded in the emotional processing structure as an external variable and serve as a major intervention measure. However, this study conducted an in-depth analysis of different types of emotional processing patterns through latent profile analysis. Compared with previous results that physical exercise directly reduces aggressive behavior, this study emphasizes more on the trend of type differentiation, reflecting the diversification and imbalance of emotional processing structures. This finding broadens the linear research trend of physical exercise intervening in emotional processing, and suggests that subsequent research should combine the person-centered development perspective, pay attention to the diversified hierarchical strategies of emotional processing types, and avoid overgeneralization.

### 4.3 Predictive effects of physical exercise and emotional processing on aggressive behavior

The above regression analysis, after controlling for demographic variables, found that the explanatory rate of physical exercise and emotional processing on aggressive behavior reached 63.6%, with a relatively stable overall model and significant predictive effect. The results further confirmed that Research Hypothesis H4 was valid: after controlling for demographic variables, physical exercise and emotional processing indicators can still significantly predict aggressive behavior. Specifically, from the data in the table, anger rumination had the largest predictive effect size on aggressive behavior, followed by expressive suppression, while cognitive reappraisal and physical exercise had significant negative predictive effects. The results are consistent with the conclusions of general aggressive behavior research and conform to the “emotion-cognition-behavior” path proposed by scholars (Larsson et al., 2024). Anger rumination increases the duration of an individual’s emotional internal friction and the probability of external aggressive behavior. Although expressive suppression controls an individual’s overt behavior in a short time, there is a possibility of an outbreak of external aggressive behavior at any time (Hofmann et al., 2024). Cognitive reappraisal reduces the definition and scope of external threats by redefining and interpreting the situation, thus reducing the probability of overt aggressive behavior (Li et al., 2023). The results of this study are consistent with existing conclusions, further clarifying the variable relationships in the standardized path coefficients and structural stratification, explaining the differences in the weights of different emotional processing strategies in aggressive behavior, and further confirming that anger rumination is the most dangerous predictive factor, which is consistent with the conclusions of previous scholars (Oliva et al., 2023).

At the same time, the finding that physical exercise can significantly and negatively predict aggressive behavior is of great significance. Previous psychological scholars have proposed that regular physical exercise can improve an individual’s self-control ability and executive power, enhance emotion regulation strategies, and reduce the occurrence of impulsive externalizing problem behaviors such as aggressive behavior and bullying behavior (Yang et al., 2022). Scholars’ Self-Control Theory also emphasizes that the improvement of an individual’s behavior inhibition ability will reduce the probability of external aggressive behavior (Kaveh et al., 2025). The results revealed a significant negative association between physical exercise and aggressive behavior. Given the cross-sectional design, the findings are merely correlational. The link between physical aggression and aggressive behavior may involve multiple pathways, yet longitudinal tracking and experimental designs are still required. Combined with the above results of the correlation between physical exercise and cognitive reappraisal, emotional rumination and other variables, it can be considered that physical exercise is embedded in the multi-structure of emotional processing and plays a unique protective role at the behavioral level. In summary, the dual-path expression mechanism further broadens the theoretical system of physical exercise intervening in aggressive behavior, suggesting that in the intervention of adolescent aggressive behavior, physical exercise should be combined with the cultivation of emotion regulation ability to form structured stratification and individualized research, so as to achieve the vision of targeted and precise services.

### 4.4 Research limitations and prospects

Although this study conducted a detailed analysis of emotional processing, physical exercise and aggressive behavior through a large-scale survey in six provinces in China and adopted latent profile analysis and regression analysis methods, there are still some limitations. First, this study adopted a cross-sectional baseline survey to explore the significance and correlation between variables, but it is not sufficient to support the causal relationship between them. There may be an interaction or interdependence between physical exercise and emotional processing, and future research needs to further demonstrate and distinguish the relationship between them through rigorous experiments. Secondly, the information of all research variables was obtained through self-report scales. Although the common method bias test in this study did not reach the critical value, the subjects’ humanistic literacy and social and cultural background will be affected by social desirability. Subsequent research needs to include other groups for dialectical analysis and sorting to increase the objectivity and comprehensiveness of the results. In addition, this study focused on cognitive reappraisal, expressive suppression and emotional rumination in emotional processing, and did not conduct a comprehensive and in-depth investigation of other emotion regulation strategies. Future research needs to comprehensively and systematically examine the effects of different emotion regulation strategies and include more emotional processing methods to increase the objectivity of the research. Finally, this study mainly investigated subjects from six provinces in China, which may have some cross-cultural differences. If funds permit, future research should include research subjects from different countries and regions to reduce biases caused by cultural background differences and further clarify the internal characteristics of adolescent aggressive behavior under different cultural backgrounds.

## 5 Conclusion

Based on a large-scale empirical survey of middle school students and latent profile analysis, this study systematically analyzed the association between physical exercise and emotion processing with aggressive behavior from the perspective of emotion processing types, and drew the following conclusions: (1) Emotional processing of middle school students presents diversified structural characteristics; emotion regulation is not a single continuous variable relationship, and the overall internal structure has heterogeneous characteristics. (2) There are significant differences in aggressive behavior and physical exercise among different emotional processing types: the more adaptive the emotional processing structure, the lower the level of aggression and the higher the participation in physical exercise. (3) After controlling for demographic variables, physical exercise and emotional processing can still significantly and negatively predict aggressive behavior; anger rumination and expressive suppression are risk factors for aggressive behavior, while cognitive reappraisal and physical exercise are important protective factors. There may be a dual-path expression mechanism in the effect of physical exercise on the expression of aggressive behavior.

## Data Availability

The raw data supporting the conclusions of this article will be made available by the authors, without undue reservation.
